# Correlation analysis between the rate of respiration in the root and the active components in licorice (*Glycyrrhiza uralensis*)

**DOI:** 10.3892/etm.2013.1387

**Published:** 2013-11-07

**Authors:** PEIJUN GUO, ZHIRONG SUN, WENLAN LIU, LONG CHEN, YUAN DU, XINXIN WEI

**Affiliations:** School of Chinese Materia Medica, Beijing University of Chinese Medicine, Beijing 100029, P.R. China

**Keywords:** *Glycyrrhiza uralensis*, root respiration, active component, correlation analysis

## Abstract

The aim of this study was to investigate the correlation between root respiration and the percentage of active components in licorice (*Glycyrrhiza uralensis* Fisch.), in order to provide a foundation for the regulation and modulation of the quality of *G. uralensis*. Respiration efflux of annual and biennial *G. uralensis* was determined using a Li-7000 CO_2_/H_2_O analyzer. The root systems were scanned at a resolution of 3,000 dpi using an Epson Expression 10000XL scanner. Root growth was determined by analyzing the scanned images using WinRHIZO version Pro2007d software and the rate of respiration in the root was subsequently calculated. In addition, the percentages of the five major active components in licorice, glycyrrhizic acid, glycyrrhizin, isoliquiritin, liquiritigenin and isoliquiritigenin, were detected using high-performance liquid chromatography (HPLC). The correlation between the root respiration and the percentage of the active components was investigated. Significant seasonal changes were observed in the rates of respiration of first and zero-class roots. In annual and biennial *G. uralensis*, the maximum and minimum values for rate of respiration were present in July (P<0.05) and November (P<0.05), respectively. The correlation coefficients between the five major active components and the rate of respiration were −0.304 (glycyrrhizin), −0.129 (liquiritigenin), −0.441 (glycyrrhizic acid; P<0.05), −0.471 (isoliquiritin; P<0.05) and 0.148 (isoliquiritigenin). The percentages of glycyrrhizic acid and isoliquiritin were significantly negatively correlated with the rate of respiration in annual and biennial *G. uralensis*. Understanding the correlation between the root rate of respiration and the active components in *G. uralensis* may be beneficial to ensuring the quality of cultivated *G. uralensis*.

## Introduction

Licorice (*Glycyrrhiza uralensis* Fisch.), an ancient medicinal herb, has been frequently used in the medical, food processing and daily chemical industries. However, wild *G. uralensis* resources have been severely damaged and, in some places, are on the verge of extinction (1). For decades, the cultivation of *G. uralensis* in China has been proposed as a substitute for the wild resources. However, several problems, including poor production and unsatisfactory root quality, have been described. In particular, the percentage of glycyrrhizic acid in the cultivated *G. uralensis* has been measured to be 0.49–1.51% (1), which is less than the standard percentage (>2%) set by the Pharmacopoeia of the People's Republic of China. Extensive studies have been performed to investigate the ability of water (2), nutrients (3) and seeding density (4) to increase the percentages of the active components in cultivated *G. uralensis*. However, no ideal conditions have been identified to enhance the percentages of the active components.

Root respiration has a significant impact on the primary productivity of plants (5). It has been demonstrated that 50–80% of the total net primary production is accounted for by the net primary production in the root (1). Högberg *et al*(6) revealed that 75% of the carbon allocated to the root was for respiration, which provided the energy for the growth, vital activity and the nutritional intake of the root (7). However, the effects of environmental and cultivation control factors on the synthesis and accumulation of the active components in *G. uralensis* remain unclear. In addition, no similar studies have been performed to evaluate the correlation between root respiration and the levels of the active components in *G. uralensis*. In this study, the aim was to analyze the fluctuations in root respiration and to investigate the correlation between root respiration and the five major active components (glycyrrhizic acid, glycyrrhizin, isoliquiritin, liquiritigenin and isoliquiritigenin) in *G. uralensis*. Glycyrrhizic acid has been widely used in the treatment of various liver diseases (8–14), while glycyrrhizin and isoliquiritin have been observed to exert antidepressive effects in mice (15). Liquiritigenin was demonstrated to prevent acute acetaminophen-induced liver injuries in rats (16) and isoliquiritigenin has been indicated to inhibit the growth and proliferation of various cancer cells and induce the apoptosis of cancer cells. In addition, isoliquiritigenin may promote the cellular proliferation of normal tissues (17). The results of this study may aid the cultivation of *G. uralensis* and promote the sustainable use of *G. uralensis* resources.

## Materials and methods

### Cultivation of G. uralensis

The *G. uralensis* seeds and seedlings were grown in Hangjingqi (Inner Mongolia, China). The plants were cultivated in April 2011 in a growth chamber in the Beijing University of Chinese Medicine (39º55′ N, 116º28′ E, at an altitude of 54.7 m). The plants were cultivated at an average temperature of 11.8ºC and an annual rainfall of 577 mm. The average evaporation capacity was 1,861 mm, while the comparative humidity was 62%.

The *G. uralensis* was grown in a polyvinyl chloride (PVC) tube, which was 5 cm above the ground. The tube was filled with sandy loam, containing organic matter (0.286%), alkali-hydrolyzable nitrogen (35.88 mg/kg), soil-available phosphorus (3.0 mg/kg), soil-available potassium (85.18 mg/kg) and CaCO_3_ (2.71%). The pH value of the sandy loam was maintained at 7.87. The diameter and length of the tube were 30 and 80 cm, respectively, and the bottom of the tube was sealed with gauze. The seeding and transplantation of the *G. uralensis* were conducted on May 10 each year. Four *G. uralensis* plants were cultivated in one PVC tube. The relative water content in the tube was maintained between 60 and 70%. No fertilizer was given to the plants.

### Determination of the rate of respiration

In order to evaluate the rate of respiration, the root was cleaned with distilled water and wrapped with wet gauze. The categorization of the root was performed as previously described (18). A zero-class root is also called an axial root and first-class roots mean the lateral roots growing on the axial root. Subsequently, the rate of respiration was calculated in accordance with the method from a previous study (19). To avoid wound respiration, Vaseline was smeared onto the section of the root system. The root was then placed in a Li-7000 CO_2_/H_2_O analyzer (Li-Cor, Lincoln, NE, USA), in which the concentration of CO_2_ released by the root system was analyzed subsequent to constant airflow being obtained. In addition, the rate of respiration was calculated based on the CO_2_ flux.

### Scanning of the root system

The root systems were scanned at a resolution of 3,000 dpi using an Epson Expression 10000XL scanner (Epson Co., Ltd., Long Beach, CA, USA). Root growth was measured by analyzing the scanned images using WinRHIZO version Pro2007d software (Regent Instruments Inc., Quebec Canada). All the experiments were performed at least six times.

### Component evaluation of the root system

The samples were oven-dried at 50ºC until a constant weight was achieved, prior to the roots being weighed and crushed. A 40-mesh screen was used as the threshold separating thick and fine roots. Subsequently, the active components of the *G. uralensis* were analyzed using a high-performance liquid chromatography (HPLC) assay with ultraviolet detection, based on a previous description (20). In brief, 5 ml *G. uralensis* (0.01 g) was added to 5 ml ethanol (70%), prior to ultrasound extraction being performed at 40ºC for 30 min (250 W). Following cooling at room temperature, 70% ethanol was added and the mixture was filtered using a Millipore filter with a diameter of 0.45 μm (Millipore, Billerica, MA, USA). A chromatographic column (Diamonsil^®^ C18, 250×4.6 mm, 5 μm; Agilent Technologies Inc., Santa Clara, CA, USA) was used for the HPLC analysis. A methyl cyanide-phosphoric acid mixture was used as the mobile phase. The wavelengths used for the analysis were as follows: λ_1_=237 nm, λ_2_=365 nm.

### Statistical analysis

SPSS 19.0 statistical software (SPSS, Inc., Chicago, IL, USA) was used for the data analysis. P<0.05 was considered to indicate a statistically significant difference.

## Results

### Changes in root respiration

**Figure 1:**
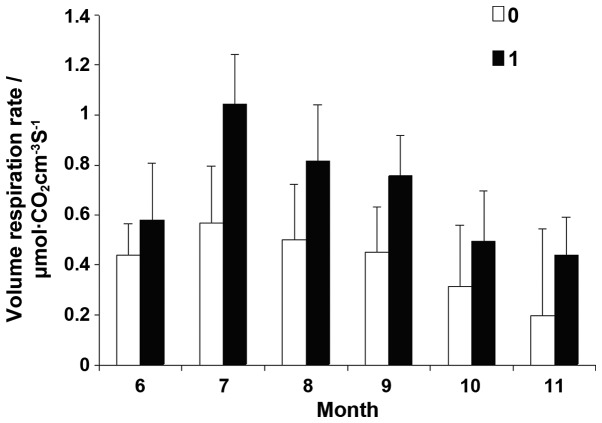
Changes in the root respiration of the annual licorice. White bars, zero-class roots; black bars, first-class roots.

**Figure 2:**
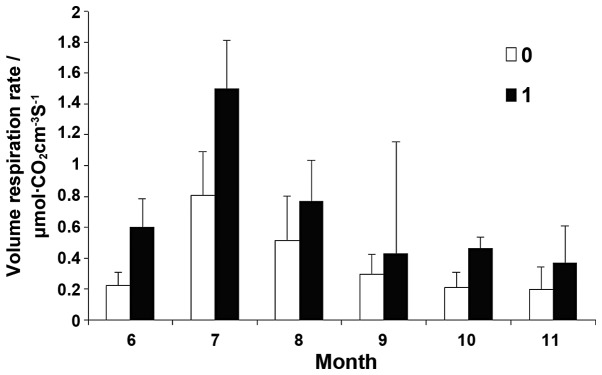
Changes in the root respiration of the biennial licorice. White bars, zero-class roots; black bars, first-class roots.

In this study, samples of annual and biennial *G. uralensis*, harvested in June and November, 2011, were used to determine the rate of respiration. A significant difference was observed in the volume rate of respiration between the annual and biennial *G. uralensis* (P<0.05, Figs. 1 and 2). In the annual and biennial *G. uralensis*, the maximum and minimum values for the rate of respiration were obtained in July (P<0.05) and November (P<0.05), respectively. Compared with the axial root, a marked increase in the rate of respiration was detected in the first-class root in the annual (0.688 versus 0.375 μmol CO_2_cm^−3^sec^−1^; P<0.05) and biennial (0.686 versus 0.411 μmol CO_2_cm^−3^sec^−1^; P<0.05) *G. uralensis* for months 6–11. These results were consistent with those obtained in a previous study by Ren *et al*(21).

The maximum rate of respiration was affected by the soil temperature, water content and nutritional intake. In the present study, higher rates of respiration were observed in July and August, which was likely to be correlated with the soil temperature and root metabolism.

### Fluctuation in active component levels

Five active components, specifically, glycyrrhizic acid, glycyrrhizin, isoliquiritin, liquiritigenin and isoliquiritigenin, were analyzed in *G. uralensis* in our study.

**Figure 3:**
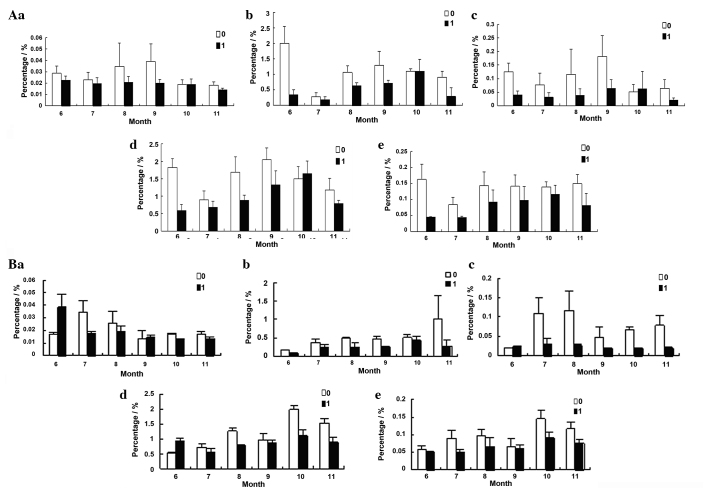
Seasonal comparisons of the five major active components in annual and biennial licorice. (A) Percentages of (Aa) glycyrrhizin, (Ab) liquiritigenin, (Ac) glycyrrhizic acid, (Ad) isoliquiritin and (Ae) isoliquiritigenin in annual licorice. (B) Percentages of (Ba) glycyrrhizin, (Bb) liquiritigenin, (Bc) glycyrrhizic acid, (Bd) isoliquiritin and (Be) isoliquiritigenin in biennial licorice. White bars, zero-class roots; black bars, first-class roots.

Fig. 3 shows the fluctuations in the percentages of glycyrrhizin, liquiritigenin, glycyrrhizic acid, isoliquiritin and isoliquiritigenin present in the *G. uralensis* harvested in June and November, respectively. The data shown in the figure indicate that significant fluctuations occurred in the percentages of the five major components (P<0.05), depending on the season. The percentage of glycyrrhizic acid measured in the axial root in the present study was consistent with the value obtained in a previous study (3). Furthermore, the maximum percentage of glycyrrhizic acid in annual *G. uralensis* was apparent in September, which was also consistent with a previous investigation (22).

As shown in Table I, the levels of the five major components in the axial roots were higher than those of the first-class roots. In addition, the percentages of glycyrrhizin, isoliquiritin, liquiritigenin and isoliquiritigenin were higher in the biennial *G. uralensis* than in the annual *G. uralensis*.

### Correlation between root respiration and the levels of the active components in G. uralensis

Table II summarizes the correlation between root respiration and the percentages of the active components in *G. uralensis*. A previous study indicated that the rate of respiration in the root increased as the nitrogen concentration in the root increased (23). In general, the first-class root exhibited the maximum content of nitrogen and rate of respiration. In plants, ~90% of nitrogen has been shown to be present in the form of various proteins (24). The levels of glycyrrhizic acid and glycyrrhizin have been demonstrated to be negatively correlated with primary metabolites, including soluble protein, in the roots (25). Therefore, compared with the axial root, a higher nitrogen content and rate of respiration and a lower secondary metabolite content were apparent in the first-class root.

## Discussion

The rates of respiration in the axial and first-class roots of annual and biennial *G. uralensis* showed significant fluctuations in the different seasons. The rates of respiration were notably higher in July and were predominantly lower in November. In the present study, it was identified that the rate of respiration was negatively correlated with the levels of glycyrrhizin, liquiritigenin, glycyrrhizic acid and isoliquiritin, while isoliquiritigenin was positively correlated with the rate of respiration. Based on these results, it may be possible to enhance the accumulation of active components by modulating the rate of respiration in *G. uralensis*. To the best of our knowledge, the synthesis and accumulation of the secondary metabolites was affected by air (26), sunlight (1), water (27), soil nutrition (28) and temperature (29). In addition, the root respiration was affected by atmospheric CO_2_ concentration (23), soil temperature (27), soil nutrition (24,25) and water content in the soil (30). In the present study, the root respiration was negatively correlated with the levels of the major active components in *G. uralensis*, demonstrating that it may be possible to alter the percentage of the active components by modulating the root respiration.

It has been demonstrated that the rate of respiration in the root is positively correlated with the root tissue nitrogen concentration (31). Therefore, the nitrogen concentration in the root may be used to evaluate the root respiration, providing greater accuracy than biomass (32). In the present study, the levels of glycyrrhizic acid and isoliquiritin were significantly correlated with the rate of respiration, with correlation coefficients of −0.441 and −0.471, respectively. This indicated that glycyrrhizic acid and isoliquiritin may be used as indices for the evaluation of root respiration.

Wild *G. uralensis* resources have been severely damaged in a number of places due to over-excavation. Although biotechniques have been proposed for the cultivation of the *G. uralensis*, the results have been controversial (33). To meet the demands for *G. uralensis*, it is crucial to identify the optimal cultivation conditions. Wang *et al*(15) suggested that agronomic measures were important for the cultivation of *G. uralensis*(15). In addition, it has been indicated that increased nutrition and water content may attenuate the percentage of glycyrrhizic acid (34). However, the correlation between the levels of active components and environmental factors remain poorly defined.

In order to expand the *G. uralensis* resources, biotechniques have been used to produce the plant. However, the results have not been consistent (35) and the techniques have not been used in practice. Our study showed that the root respiration was significantly negatively correlated with the levels of glycyrrhizic acid and isoliquiritin. In addition, the root respiration may affect the synthetic pathway of active components in *G. uralensis*.

## Figures and Tables

**Table I:** Percentages of the active components in *G. uralensis* root.

*G. uralensis*	Root system	Glycyrrhizin (%)	Liquiritigenin (%)	Glycyrrhizic acid (%)	Isoliquiritin (%)	Isoliquiritigenin (%)
Annual	0	0.50	0.07	1.61	0.09	0.02
	1	0.26	0.02	0.86	0.06	0.01
Biennial	0	1.11	0.10	1.52	0.13	0.03
	1	0.54	0.04	0.98	0.08	0.02

**Table II:** Correlation between root respiration and the content of the active components in *G. uralensis.*

Component	Growth year	Fitting equation	R^2^	r
Glycyrrhizin	Annual *G. uralensis*	Y=−0.2461x+0.5166	0.1409	−0.375
	Biennial *G. uralensis*	Y=−1.1270x+1.4478	0.2350	−0.485
	Mixture	Y=−0.4695x+0.8611	0.0921	−0.304
Liquiritigenin	Annual *G. uralensis*	Y=−0.0031x+0.0500	0.0010	−0.032
	Biennial *G. uralensis*	Y=−0.0620x+0.1070	0.0932	−0.305
	Mixture	Y=−0.0183x+0.0706	0.0167	−0.129
Glycyrrhizic acid	Annual *G. uralensis*	Y=−0.5873x+1.3244	0.2680	−0.518
	Biennial *G. uralensis*	Y=−0.9622x+1.7805	0.2016	−0.449
	Mixture	Y=−0.6793x+1.4993	0.1942	−0.441^a^
Isoliquiritin	Annual *G. uralensis*	Y=−0.0353x+0.0995	0.2030	−0.451
	Biennial *G. uralensis*	Y=−0.1226x+0.1754	0.4811	−0.694^a^
	Mixture	Y=−0.0582x+0.1259	0.2215	−0.471^a^
Isoliquiritigenin	Annual *G. uralensis*	Y=0.0063x+0.0169	0.0739	0.272
	Biennial *G. uralensis*	Y=−0.0028x+0.0247	0.0739	−0.086
	Mixture	Y=0.0039x+0.0196	0.0218	0.148

a P<0.05.
